# Posttraumatic Stress Disorder and Related Disorders among Female Yazidi Refugees following Islamic State of Iraq and Syria Attacks—A Case Series and Mini-Review

**DOI:** 10.3389/fpsyt.2017.00282

**Published:** 2017-12-13

**Authors:** Inga Gerdau, Jan Ilhan Kizilhan, Michael Noll-Hussong

**Affiliations:** ^1^Department of Psychosomatic Medicine and Psychotherapy, University of Ulm, Ulm, Germany; ^2^Duale Hochschule Baden-Württemberg Villingen-Schwenningen, Villingen-Schwenningen, Germany

**Keywords:** Yazidi, posttraumatic stress disorder, abuse history, mental disorders, somatic symptom disorder, transcultural psychiatry

## Abstract

Following the severe attacks by the so-called “Islamic State of Iraq and Syria” on the Yazidi population, which started in summer 2014, the state government of Baden-Württemberg, Germany, funded a Special-Quota Project to bring 1,000 very ill or left-behind women and children who were being held hostage to 22 cities and towns in Baden-Württemberg to receive integrated care. Here, we report for the first time on the cases of four Yazidi women living in Ulm, Germany, focusing on the clinically observed and psychometrically assessed mental phenomena or disorders. Our primary aim was to explore what International Classification of Diseases, 10th Revision diagnoses are present in this population. Although highly traumatized, these women were suffering primarily from adjustment disorder rather than posttraumatic stress disorder according to official classification systems. Despite their symptoms of depression and anxiety, the women’s responses to self-assessment questionnaires provided no evidence of compulsion, somatization, or eating disorders. The results suggest that further investigation of the individual-level effects of rape and torture, as well the historic, systemic, and collective effects, e.g., on families and societies, is required.

## Background

Psychiatric symptoms and mental disorders among refugees are recognized as an urgent problem (1–5), especially in the Yazidi population (6, 7) after the severe attacks by the so-called “Islamic State of Iraq and Syria” (ISIS) in July 2014 (8, 9). Recently, it has been shown that about 43% of the victims of these attacks met the DSM-IV diagnostic criteria for posttraumatic stress disorder (PTSD), about 40% met the criteria for major depressive disorder (MD), and about 26% met the criteria for both disorders. A higher proportion of women than men suffered from PTSD and MD, and women with PTSD or depression were more likely than their male counterparts to report having experienced or witnessed the death of a spouse or child. Women with PTSD reported the core symptoms of PTSD (flashbacks, hypervigilance, and intense psychological distress due to reminders of trauma) more frequently than their male counterparts, and women tended to show undermodulation of emotions and low self-esteem in response to traumatic stress (10). Depressed women were more likely than their male counterparts to report feelings of guilt (11) or worthlessness (10). Severity of posttraumatic symptoms seems to be the strongest predictor of impaired health-related quality of life in PTSD outpatients (12). Until now there have been no published observational or clinical studies of the time course or long-term effects of these complex psychopathological phenomena and the associated risk and resilience factors (13–16) including cultural and anthropological factors (3), especially in the comparatively rare case of Yazidi patients given refuge in a “safe” Western country (17, 18).

We visited four Yazidi women (Table 1) who had been living in the city of Ulm, Germany since December 2015, as part of a special-quota project in the German region of Baden-Wuerttemberg that was designed to support some of the estimated 2,500 women and children who had been held hostage by ISIS (19). In Germany, they received permanent residence permit, psychological and social care and attended a language course. Initially, all the women were housed in an urban refugees’ home for women with dependent children and were—where appropriate —offered the opportunity to take part in stabilizing, non-verbal, supportive art therapy. The impressions of the trained art therapist and other members of the caring staff were that the women presented in this case report needed psychosomatic support from our cooperating university clinic as they suffered badly from diverse physical, social, and psychological problems. Before being assigned to further treatment, all the women were reassessed to ensure that a valid, up-to-date, clinical diagnosis was available and thus avoid causing further harm. A trained German, female psychotherapist (IG) carried out psychodiagnostic interviews (one to three sessions, ~60 min each) in our outpatient clinic. A female translator accompanied the women to these interviews and assisted them, e.g., in responding to self-assessment questionnaires, as none of them spoke German or English and/or were unable to read at the time of the initial interviews (20).

**Table 1:** Demographic and historical profiles of the Yazidi women.

	Age in years in 2015	Education (years)	Marital status	No. of children	Victim of sexual violence	Victim of physical violence	Captivity	Diseases and injuries	Suicidal tendency
A	45	8	Married	5	No	Yes	Yes	Slipped disk	No
B	27	1	Single	0	Yes	Yes	Yes	None	No
C	40	1	Single	0	Yes	Yes	Yes	None	No
D	35	None	Married	9	No	Yes	Yes	None	No

To our methods: First, all women underwent preclinical, face-to-face examinations (one session, up to 90 min, 60 min on average) by a Kurdish-German psychological psychotherapist (Jan Ilhan Kizilhan) who speaks Kurdish (Kurmanici) in conjunction with a psychometric screening in Northern Iraq (between March and December 2015) before they emigrated in December 2015. Approximately 6 months later, four of them underwent the abovementioned comprehensive clinical examination in Germany (completion: July 2016) to confirm preliminary diagnoses in accordance with established national standards before any therapeutic procedures were administered. The screening in Iraq comprised the ICD-10-Symptom-Rating 2.0 (21, 22), the Essen Trauma Inventory (23, 24), the Impact of Event Scale—Revised (25–27), and the Beck Depression Inventory II (BDI-II) (28–34) (Table 2). In Germany, they first underwent a standard medical examination (35) and then psychiatric assessment by a certified, supervised female psychotherapist using the Mini-International Neuropsychiatric Interview (German Version 6.0.0) (36) to give a general overview of current psychological state, the PTSD section of the structured clinical interview for DSM-IV (SCID-PTSD) (37, 38) to provide a more detailed picture of possible trauma symptoms and, once again, the BDI-II (Tables 2 and 3; Table S1 in Supplementary Material). The Global Assessment of Functioning, which yields a numeric score [0 (severely impaired)–100 (extremely high functioning)] was also used to rate the women’s social, occupational, and psychological functioning (39, 40). The Global Assessment of Relational Functioning (41–43) was also estimated (completion: July 2016). All patients gave written informed consent for publication.

**Table 2:** Summary of psychometric scores and diagnoses [International Classification of Diseases, 10th Revision (ICD-10)] received in 1 year.

Patients	May 2015, Duhok, Iraq					July 2016, Ulm, Germany			

	ETI^a^	IES-R^b^	ISR total score^c^	BDI-II^d^	ICD-10 (clinical exam)	BDI-II^d^	GAF	GARF	ICD-10-GM (clinical exam + M.I.N.I. + SCID-PTSD)
A	47	1.09	0.61	28	F32.1, F43.1 (tentative diagnosis)	10 (↓)	55	78	F42.23, Z60.5, Z63.4, Z73.3, Z91.4
B	53	1.15	1.03	34	F43.1	5 (↓)	65	80	F42.23, Z60.5, Z63.4, Z73.3, Z91.4
C	50	0.39	1.02	24	F43.1	1 (↓)	61	76	F42.23, F43.8, T74.8, Z60.5, Z63.4, Z73.3, Z91.4
D	41	−0.1	0.55	15	F32.1, with traumatic symptoms	20 (↑)	48	71	F42.23, Z60.5, Z63.4, Z73.3, Z91.4

*^a^0–15: normal; 16–26: marginal, probable partial PTSD; >27: PTSD*.

*^b^x > 0 indicates that PTSD is very probable*.

*^c^≥0.6 minimal symptoms; ≥0.9 modest symptoms; ≥1.7 severe symptoms*.

*^d^0–13: minimal symptoms of depression; 14–19: mild symptoms of depression; 20–28: moderate symptoms of depression; 29–63: severe depression*.

*ETI, Essen Trauma Inventory; IES-R, Impact of Event Scale—Revised; ISR, ICD-10-Symptom-Rating; BDI-II, Beck Depression Inventory II; GAF, Global Assessment of Functioning; GARF, Global Assessment of Relational Functioning; PTSD, posttraumatic stress disorder; M.I.N.I., Mini-International Neuropsychiatric Interview*.

*ICD-10-GM-Codes: F42.23 = adjustment disorder with mixed anxiety and depression; F43.8 = other reactions to severe stress; T74.8 = abuse of a person not otherwise classified; Z60.5 = target of perceived adverse discrimination and persecution; Z63.4 = disappearance and death of family members; Z73.3 = stress, not otherwise classified; Z91.4 = other personal history of psychological trauma, not otherwise classified*.

*Severe symptoms are marked in red*.

**Table 3:** ICD-10-Symptom-Rating (International Classification of Diseases, 10th Revision) subscale scores in May 2015, Iraq.

	Depressive syndrome^a^	Anxiety syndrome^a^	Compulsive–obsessive syndrome^b^	Somatoform syndrome^c^	Eating disorder syndrome^d^	Supplementary items^e^
A	2.0	0.25	0	0	0	1.0
B	2.75	1.0	0	0	0	1.73
C	2.5	1.0	0	0	0	1.82
D	1.75	0.25	0	0	0	0.91

*^a^Severity of symptoms: ≥0.75: suspicious; ≥1: marginal; ≥2: modest; ≥3: severe*.

*^b^Severity of symptoms: ≥0.67: suspicious; ≥1: marginal; ≥2: modest; ≥3: severe*.

*^c^Severity of symptoms: ≥0.33: suspicious; ≥0,75: marginal; ≥1.25: modest; ≥2.67: severe*.

*^d^Severity of symptoms: ≥0.33: suspicious; ≥0.67: marginal; ≥1.5: modest; ≥2.67: severe*.

*^e^The supplementary items do not represent an individual construct. They comprise (a) items that can be ascribed to several syndromes and (b) screening items, which can be used as to guide a more comprehensive search for impairment in the form of specific syndromes. All items attracting a score of ≥1 should be explored more closely (44)*.

## Case Presentations

### Patient A

Patient A (46 years old) reported that she and her family had been attacked by ISIS in August 2014. She became separated from her husband and her four older children. She and her youngest son managed to escape, but the rest of the family was kidnapped. Her daughters spent 14 months in captivity before Patient A was able to ransom them. Patient A, her three daughters and her youngest son were living together in Germany at the time of interview, but her husband and oldest son were still captives of ISIS. This was her main concern. She also felt guilty because it had been she who chose the route home on the day the family was attacked by ISIS fighters. She ruminated and cried a lot. A reported that she was very nervous and agitated on some days, while on others she was totally exhausted and had no energy. Earlier on she had enjoyed having friends come over to her house and cooking for them, but at time of the interview she had no interest in doing things like that and had withdrawn from social events.

In accordance with the psychometric outcomes and the clinical examination the following differential International Classification of Diseases, 10th Revision (ICD-10) (45–48) diagnoses were made:

– adjustment disorder (AD) with mixed anxiety and depression (F42.23);
– target of perceived adverse discrimination and persecution (Z60.5);
– disappearance and death of family members (Z63.4);
– stress, not otherwise classified (Z73.3);
– other personal history of psychological trauma, not otherwise classified (Z91.4).

### Patient B

Patient B was 23 years old on the day of investigation. Like Patient A, she and her family had been attacked and kidnapped by ISIS in August 2014. Men and women had been separated. Patient B and her three sisters had been part of a group of 100 women displaced to Mosul. There she was forced to live with ISIS combatants, who hit and raped her. In April 2015, her uncle was able to buy her and her sisters’ release, but her parents remained in captivity. Patient B said she did not know if they were still alive. She suffered from flashbacks, especially to the maltreatment she had received. As her flashbacks were more intense in the evening, when she went to bed, Patient B slept badly. Patient B also had problems concentrating. She found it very helpful to talk about the things she had experienced and to cry.

In accordance with the psychometric outcomes and the clinical examination the following ICD-10 diagnoses were made:

– AD with mixed anxiety and depression (F42.23);
– target of perceived adverse discrimination and persecution (Z60.5);
– disappearance and death of family members (Z63.4);
– stress, not otherwise classified (Z73.3);
– other personal history of psychological trauma, not otherwise classified (Z91.4).

### Patient C

Patient C was 40 years old at the time of interview. Her village had also been attacked by ISIS in August 2014. The men and older women were killed on the spot. The younger women were kidnapped and sold. Patient C reported that she was bought by a high-ranking ISIS military leader in Iraq. She spent most of her time captivity confined to a cell and only sometimes had the company of another Yazidi woman. Patient C believed that some sort of tranquilizing drug had been added to the captives’ food, because she remembered sleeping for abnormally long periods. She was raped several times a week. After nine months she was sold to another man in Mosul, where she was once again sexually abused. In September 2015, she managed to flee with the help of her uncle. Her parents, two of her brothers and her aunt remained captives of ISIS and she did not know if they were still alive. Patient C reported that she suffered from flashbacks and bad dreams revolving around her family. At the time of the interview, she had talked only to professionals about the things that had happened to her. She had avoided talking to other Yazidi woman in order not to stress them, knowing that they had had similar experiences. She explained that when other women started talking about ISIS she would leave the room. She was no longer interested in the things she used to like, such as visiting friends, drawing, and sewing. She said that she had difficulty concentrating, mainly because she was always thinking about her parents. Despite everything, Patient C stated that she was still optimistic and had a positive view of the future. She hoped to be able to start a family.

In accordance with the psychometric outcomes and the clinical examination, the following ICD-10 diagnoses were made:

– AD with mixed anxiety and depression (F42.23);
– other reactions to severe stress (ICD-10: F43.8);
– abuse of a person not otherwise classified (T74.8);
– target of perceived adverse discrimination and persecution (Z60.5);
– disappearance and death of family members (Z63.4);
– stress, not otherwise classified (Z73.3);
– other personal history of psychological trauma, not otherwise classified (Z91.4).

### Patient D

Patient D (34 years old) lived in the refugees’ home with her nine children and her husband. She was the only woman whose husband had managed to get to Germany. We saw Patient D three times, because she needed to talk and suffered a lot. Patient D and her family had lived near Mosul. She was working on a farm when ISIS invaded her village. The terrorists built fortresses around the village, and Patient D and the other inhabitants were forced to grow fruits and vegetables for the ISIS combatants. Patient D was 14 weeks pregnant when the village was invaded and because she was living under constant threat and in fear, she lost the baby. Patient D and her husband hid their two teenage daughters in a hole in the ground to try to protect them from being raped. Everyone in Patient D’s family carried a lethal pill that could be used to commit suicide in the event of capture by ISIS. Patient D and her husband had often thought about committing suicide as a family, but in the end, they always decided against it, because of their children and because they hoped to be rescued. Since she came to Germany, Patient D had suffered a multitude of symptoms. She felt depressed and suffered from fear, flashbacks, disordered sleep, and severe headaches. She reported feeling permanently tired and exhausted. She said she had to force herself to do even the smallest things. She would not leave the apartment on her own; she had to be accompanied by a family member at all times. Men with dark beards reminded her of the “disgusting, dirty, and smelly IS [Islamic State] men.” When she heard news stories about terror, she was shocked and stopped feeling safe in Germany. At our first meeting, Patient D reported that she got annoyed very quickly and that she often “lost control of herself.” It appeared that she sometimes hit her children; this is something she had not done before her experiences with ISIS. She knew that it was seriously wrong but could not help herself. We talked to her about how to cope with anger and fury, and as early as our second session she reported that she was managing to avoid hitting her children when she was irritated.

In accordance with the psychometric outcomes and the clinical examination, the following ICD-10 diagnoses were made:

– AD with mixed anxiety and depression (F42.23);
– target of perceived adverse discrimination and persecution (Z60.5);
– disappearance and death of family members (Z63.4);
– stress, not otherwise classified (Z73.3);
– other personal history of psychological trauma, not otherwise classified (Z91.4).

## Discussion

Refugees are a highly traumatized group of patients who present many clinical challenges, partly because many have experienced trauma related to long civil wars, torture, and ethnic cleansing The diagnosis commonly associated with the initial effects of trauma is PTSD with or without comorbid depression; however, psychosis and neurocognitive disorders are also prevalent (49). Our adult case series both confirms and extends earlier research showing that Yazidi refugee children and adolescents do not just suffer from PTSD but from various other problems such as ADs (8, 50). Patra and Sarkar noted that “adjustment disorder is a diagnostic category characterized by an emotional response to a stressful event. It is a state of subjective distress and emotional disturbance, which arises during the course of adapting to stresses of significant life changes, stressful life events, serious physical illness, or possibility of serious illness. […] When coping mechanisms fail to ameliorate stress effectively, AD is precipitated. At a variance from the largely atheoretical model of ICD-10 and DSM-IV-TR, AD is one of the few disorders that take into account the potential cause of the disorder. AD is a psychiatric diagnosis that falls between normal behavior and the major psychiatric disorders and thus produces taxonomical and diagnostic dilemmas (51)” (52). In contexts such as ours Maercker et al. (53) proposed the application of “a new diagnostic model that describes ADs as particular forms of stress response syndrome, in which intrusions, avoidance of reminders and failure to adapt are the central processes and symptoms” (53, 54).

It should also be recognized that survivors of torture have problems that extend beyond PTSD symptoms. There should be recognition of the contextual factors associated with being a (torture) survivor—including being an asylum seeker or refugee and membership of a special ethnic group (55)—and their impact on psychological and social health (4). It is possible that the evaluation process itself may have a beneficial effect on survivors’ emotional well-being, e.g., by helping the survivor understand the necessity of telling her story, illuminating the often poorly understood link between ongoing emotional suffering and past torture, strengthening the development of cognitive and emotional control, and healing the wounds of fear, humiliation, marginalization, and mistrust (56). Long-term studies indicate that, in addition to mental disorders, medical disorders such as diabetes and hypertension are also prevalent in traumatized refugees and so it is important that they receive a thorough medical evaluation that includes, e.g., the measurement of blood pressure and diabetes testing (49).

When assessing and treating outpatients from a refugee background with multiple or complex trauma (57) both the psychopathological symptoms and positive changes should be addressed (2). For example, it has been shown that trauma-focused therapy is not appropriate for all refugee children (58) although the majority of studies of traumatized refugees suggest that trauma-focused therapy reduces trauma-associated symptoms (59). There is evidence that cognitive-behavioral therapy (CBT) (60) and narrative exposure therapy (NET) are appropriate treatments for certain groups of refugees (61). Pharmacotherapy with venlafaxine and sertraline had a similar impact on PTSD symptoms when used in combination with psychotherapy (62). Intervention studies often have methodological limitations such as the absence of a control group, lack of randomization, and use of small samples (63). Furthermore, details of treatment satisfaction or adverse events are not available immediately after therapy or in the medium term. Often inadequate attention is paid to the cultural appropriateness and psychometric properties of interventions and inappropriate measures used to assess their effects. All this means that the findings should be interpreted with caution. That said, the available low-quality evidence suggests that psychological therapies have no immediate effects on posttraumatic symptoms, distress or quality of life in torture survivors although NET and CBT have been shown to produce moderate reductions in distress and PTSD symptoms over the medium term (six months after treatment). To date, there is no evidence on whether reductions in symptoms are associated with improvements in quality of life, participation in community life or social and (family) relationships in the medium term (4).

The availability of culturally sensitive services —for instance, so-called “transcultural psychiatry”—for migrants remains inadequate (64). In this context, one may speculate that the universal construct validity (65) of the diagnoses “PTSD” (66) or “complex PTSD” (67) needs further investigation (68), especially in cultures and populations like the Yazidi. Health services should consider how culture affects the expression of psychological and somatic symptoms and, e.g., avoid drawing a dualistic distinction between somatic and psychological expressions of pain (26, 69). Lack of interpreter services can have serious consequences for patients with mental disorders. Trained professional interpreters and bilingual health-care providers have a beneficial effect on patients’ satisfaction with care, quality of care and patient outcomes. Training in communication of medical information *via* an interpreter is still under development (70, 71). The difficulties of working with an interpreter highlight the need for both interpreters and (mental) health professionals to receive training in cross-language empathy and should encourage the use of transcultural models of psychotherapy and psychiatry. Some of the difficulties associated with adopting traditional, humanistic models of empathy, which tend to centralize the therapist’s perspective within empathic processes when working with interpreters, are still under debate (72).

Future research should address the aforementioned evidence gaps, involve larger samples wherever possible (4) and, among others, explore the correlating neurobiological backgrounds (73). Refugees are generally accepting psychiatric treatment if it is wisely chosen and managed, e.g., through individual evaluation and personalized treatment plans, and it can give them relief from symptoms associated with the massive trauma they have experienced (49).

## Conclusion

Refugees are a highly traumatized population, but they do not just suffer from PTSD. ADs are particular forms of stress response syndrome, in which avoidance, intrusions, and failure to adapt are the central processes and symptoms. From a clinical perspective and against the disputable universal construct validity of the PTSD diagnosis, culturally sensitive diagnostic as well as therapeutic services and caregiver or translator education are a prerequisite for achieving improvements in quality of life in this special patient population, which faces multiple problems on many levels and needs to be protected from further damage, e.g., in form of retraumatization or xenophobia. There is also a need for further investigation into the individual-level effects of rape and torture and effects on the historic or systemic/collective level, e.g., on families and society.

## Ethics Statement

Only clinical case reports are presented, therefore no accordance with the recommendations of an ethics committee was necessary. All subjects gave written informed consent for publication of this case report and any accompanying information or images.

## Author Contributions

IG examined the patients and analyzed the clinical and psychometric data in Germany. JK examined the patients and analyzed the clinical and psychometric data in Iraq. MN-H supervised IG, planned the pilot study, and was a major contributor to the writing of the manuscript. All the authors read and approved the final manuscript.

## Conflict of Interest Statement

The authors declare that the research was conducted in the absence of any commercial or financial relationships that could be construed as a potential conflict of interest. The reviewer TH and handling editor declared their shared affiliation.
