# Supplementary material for: Posttraumatic Stress Disorder and Related Disorders among Female Yazidi Refugees following Islamic State of Iraq and Syria Attacks—A Case Series and Mini-Review
Source: Front Psychiatry. 2017 Dec 13;8:282. doi: 10.3389/fpsyt.2017.00282 (PMC5733480; doi:10.3389/fpsyt.2017.00282)
Supplement: Supplementary file 1 [file Table_1.DOCX]

**Supplementary Table S1: Supplementary ISR items (ICD-10) (Tritt et al., 2013) in May 2015, Iraq**

| **ISR Items** | **Case A** | **Case B** | **Case C** | **Case D** |
| --- | --- | --- | --- | --- |
| *“I have difficulty concentrating” (ICD-10 FO)* | 1 | 2 | 1 | 1 |
| *“I think about committing suicide” (ICD-10 Indicative of depression)* | 0 | 1 | 0 | 0 |
| *“I have problems sleeping” (ICD-10 F51)* | 1 | 2 | 1 | 1 |
| *“My appetite is diminished” (ICD-10 Indication of depression and other disorders)* | 1 | 1 | 1 | 0 |
| *“I keep forgetting things” (ICD-10 F0 and stress)* | no answer | no answer | no answer | no answer |
| *“I suffer from recurring dreams or flashbacks of horrible events” (ICD-10 F43.1)* | 3 | 2 | 3 | 2 |
| *“I have mental difficulties due to intense everyday stress (such as being seriously ill, losing my job or separating from my partner, ICD-10 F43.2)* | 2 | 2 | 3 | 2 |
| *“I no longer perceive my feelings and experiences as my own” (ICD-10 F48.1)* | 2 | 3 | 2 | 1 |
| *“The people and environment around me suddenly appear unreal, distant, and lifeless to me” (ICD-10 F48.1)* | 1 | 3 | 2 | 0 |
| *“I have difficulty engaging in sexual activities” (ICD-10 F52)* | 0 | no answer | 4 | 2 |
| *“I’ve changed significantly over the past years after having experienced an extremely stressful event (such as a head injury, war or abuse, ICD-10 F62)* | 0 | 0 | 0 | 1 |
| *“My sexual preferences cause me distress” (ICD-10 F65/F66)* | 0 | 0 | 1 | 0 |
